# Medication Purchases Are Associated With the Number of Dental Treatments

**DOI:** 10.1002/cre2.70121

**Published:** 2025-05-23

**Authors:** Freja Frankenhaeuser, Håkan Källmén, Jukka Meurman, Esa Korpi, Birgitta Söder

**Affiliations:** ^1^ Department of Oral and Maxillofacial Diseases University of Helsinki Helsinki Finland; ^2^ Uppsala University Uppsala Sweden; ^3^ Department of Pharmacology University of Helsinki Helsinki Finland; ^4^ Department of Dental Medicine Karolinska Institutet Solna Sweden

**Keywords:** dental appointments, hyposalivation, medication purchase, polypharmacy, xerostomia

## Abstract

**Objectives:**

Little is known whether the number of systemic medications, indicating worsened general health, affects the number of dental appointments needed. The hypothesis is that patients purchasing more systemic medications would have an increased number of dental appointments and respective need for treatments than patients who do not purchase as many medications.

**Materials and Methods:**

Our cohort consists of 1495 participants from the Stockholm area, Sweden, initially examined in 1985. Using national population and patient registers (2005–2017), the association between the number of medication purchases and dental appointments was analyzed. The Anatomical Therapeutic Chemical Classification System for Medicines (ATC) was used. Descriptive statistics, chi‐square tests, and logistic regressions were used with several covariates like gender and socioeconomic status.

**Results:**

Purchases above the median of all medications showed a statistically significant association with more dental appointments and respective treatments. Most of the ATC system's main drug categories were significantly associated with more dental appointments, even when adjusting for covariates. Purchases of medications from 32 different ATC subgroups were significantly associated with the number of dental appointments above the median.

**Conclusion:**

In the group of adult Swedes who were studied, it was found that increased purchasing of nearly all types of drugs was associated with an increased number of dental appointments between the study years 2005–2017.

## 1 Introduction

Multimorbidity, simultaneously having two or more chronic systemic conditions, is expected to rise as the population ages (WHO 2007). Multimorbidity is associated with decreased quality of life, higher healthcare costs, and a higher risk of mortality (Makovski et al. 2019; Tooth et al. 2008). Importantly, systemic diseases and oral health are closely connected (Tavares et al. 2014). Polypharmacy, a long‐term use of more than five different medications, is also an emerging problem (Masnoon et al. 2017), which is associated with reduced saliva secretion and dry mouth (Johanson et al. 2015; Villa and Abati 2011). Polypharmacy and multimorbidity are often seen in the same patients, where also a risk appears for drug‐drug and drug‐disease interactions (Daunt et al. 2023).

Globally, 3.5 billion people suffer from oral diseases, them being the most prevalent health problem with 2 billion untreated caries lesions and 1 billion severe periodontal disease cases (Jain et al. 2024). Gingivitis, periodontitis, and periapical periodontitis are diseases associated with systemic health (Noites et al. 2022; Sebring et al. 2022; Herrera et al. 2023). Dental caries and periodontal diseases tend to accumulate in the same subjects and the same teeth (Stangvaltaite‐Mouhat et al. 2024; Mattila et al. 2010). Common denominators are the lack of oral hygiene, hyposalivation, and altered saliva secretion and their combinations. Dry mouth, an umbrella term for hyposalivation, reduced saliva secretion, low salivary flow rate, salivary gland hypofunction, and xerostomia (the term for the subjective feeling of dry mouth), are caused by hundreds of medications, psychological factors, radiation to the head and neck, as well as by systemic diseases, such as Sjögren's syndrome and diabetes (Ito et al. 2023). Hyposalivation and xerostomia can lead to decreased quality of life, impair chewing capacity, increase the risk of caries, tooth loss, candidiasis, and decrease overall well‐being (Barbe 2018; Turner 2016). In particular, hyposalivation and xerostomia are closely connected to the use of many systemic medications, such as anticholinergics, antidepressants, antihistamines, antihypertensive, and antipsychotics (Villa et al. 2016).

With the background above, our aim of the present register study was to assess the association between the number of purchasing of systemic medications and the risk of an elevated need for more dental appointments with subsequent treatments. Our hypothesis was that patients who purchased many medicines also had worse oral health, thus having more treatment needs when compared with the patients who needed fewer systemic medications.

## 2 Materials and Methods

**Figure 1:**
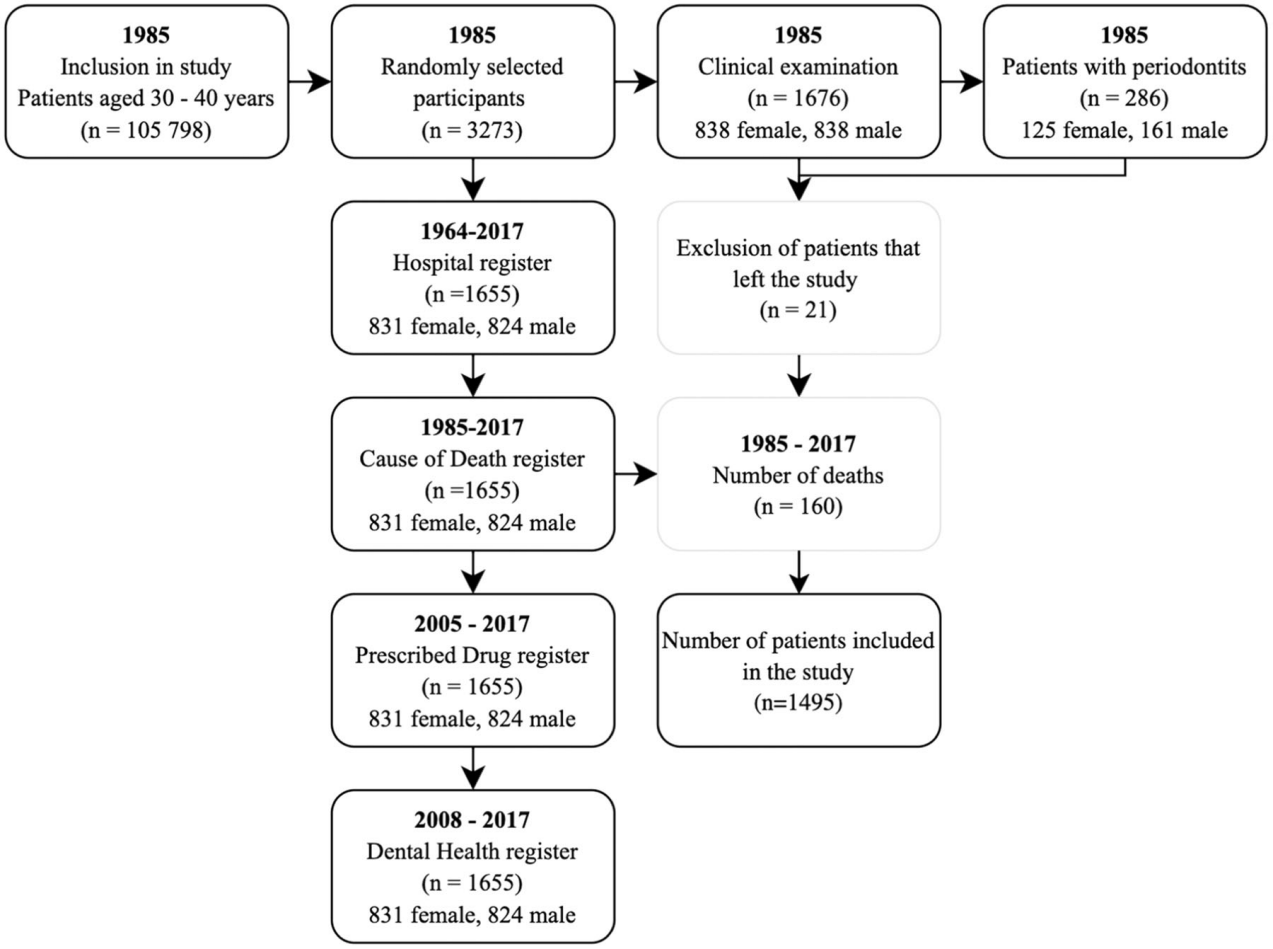
Flowchart of the present study, inclusion, and exclusion of patients, and the registers used.

The participants of the study stem from an original cohort of 105,798 individuals from the metropolitan Stockholm area, Sweden. Out of the cohort, 3273 participants were randomly selected from the individuals born the 20th day of each month and born in the years 1945‐1954 (Söder et al. 1994). In 1985, 1676 patients out of the 3273 selected patients were clinically examined at baseline. Out of the 1676 patients, a dropout of 21 patients resulted in 1655 participants. Another 160 patients were deceased before 2017 and were excluded from the analyses, resulting in 1495 patients in the present study in 2024 (Figure 1). At the time of the current study, the participants were aged 50–73 years.

The pharmacology database of the Swedish National Prescribed Drug register spans from the years 2005 to 2017, as used in the present investigation. The register includes all the medications purchased among the 1434 patients here included (Figure 1). 61 patients did not purchase any medications. The register consists of the participants´ total purchases of medications at Swedish pharmacies, excluding medical supplies and extempore medications. In total 469,789 purchases had been registered. For analyses, the medications were classified according to the Anatomical Therapeutic Chemical classification system (ATC) first in the main groups (the first anatomical level of ATC groups), and, secondly, in the subgroups (the 2nd level of the ATC groups). The ATC contains 14 main groups but in this study 13 of them were included. The ATC group “Various” (code V) was excluded due to its heterogeny and because of too few purchases recorded. Dichotomized variables were coded so that patients purchasing more medications than the median were coded as 1 (= high) and those with median or fewer purchases were coded as 0 (= low).

The dental health register used for the study is maintained by The National Board of Health and Welfare (Socialstyrelsen*)*, Sweden, and it contains data from the years 2008–2017. Included in the register are the date of the appointment, permit code for diagnosis, and code for treatments given at an appointment.

Different variables for dental treatments were created using The National Board of Health and Welfare's code for treatments. The different variables included dental exams, radiographs, saliva tests, codes for biopsy and laboratory, caries prophylaxis treatments, periodontitis treatments, extractions, endodontic treatments, dental fillings, and complete dentures. All variables were created by summarizing the number of specific treatment codes for each patient and then dichotomized based on treatments below or equal to the median (code 0 = low) or above the median (code 1 = high). A variable for the total number of dental appointments was created by summarizing the total number of dental appointments for each patient. In conformity with the summation of treatments being above the median (high = 1) or below or equal to the median (low = 0) another dichotomized variable was then created. Dental diagnoses from the register were deemed too imprecise and thus excluded from the present study. This was due to the large number of different dental providers included in the registry.

In 1985, the periodontitis diagnosis was based on the existence of at least three PD values of ≥ 5 mm in three different nonadjacent teeth. At the clinical examinations, periodontal pocket probing depths (PD) were measured at six sites per tooth, rounded to the nearest millimeter (Söder et al. 1994). Furthermore, at baseline in 1985, the patients were divided into higher or lower socioeconomic classes based on income and level of education. Patients with a lower level of education and low or no income were coded as having lower socioeconomic status (Code 1), and the ones with greater income and a higher level of education were coded as high (Code 0). In the course of the follow‐up of the original cohort, a change had been made in Sweden in the way the higher income was officially reported. Consequently, a reliable follow‐up variable of the socioeconomic status of our participants could not be calculated from the 2017 socioeconomical register of the National Board of Health and Welfare.

The socioeconomic status parameter, at baseline, was used as a covariate in this study to analyze how socioeconomic status could affect oral health. In 1985, there was no significant difference between the genders of participants with lower socioeconomic status (women *n* = 137, 17.9%, men 155, 20.3%). Since both oral health and usage of medications have been shown to differ between genders, a covariate for gender was created, where women were coded as 0 and men as 1. There were 730 men (48.8%) and 765 women (51.2%) among the 1495 participants.

The patients´ usage of tobacco products, smoking or using Swedish snus, was registered at baseline in 1985. A dichotomized tobacco consumption variable was created. Patients who used tobacco products were coded as 1 and those not using tobacco products were coded as 0.

Visual Studio Code 2, Python 3.9.10 64‐bit was used for data reorganization, summation of the registers, and calculations. For the descriptive statistics, the genders and their basic characteristics were compared by frequencies, *t*‐tests, and Chi‐square tests. A significance was set at 0.05. For medication purchases and dental appointments, the mean, standard deviation (SD), and median were calculated using frequencies. To assess the differences between high and low medication purchasers regarding the number of dental appointments Chi‐square tests were employed. Medication purchases for the main ATC groups (1st level) and the ATC subgroups (2nd level), respectively, versus high and low dental appointments were analyzed. To eliminate error due to confounders, logistic regression analyses were then conducted with covariables such as gender, tobacco usage, socioeconomic status at baseline in 1985, and further, if the participants had a hospital diagnosis before 1985 (excluding trauma and childbirth). The statistical analyses were conducted in SPSS 28.0 software.

### 2.1 Ethical Approval

The Ethics Committee of the Karolinska University Hospital at Huddinge had approved the study (Dnr 2007/1669–31; 2012/590–32; 2017/2204–32).

## 3 Results

In this study, between the years 2005–2017, of the participants 93.9% purchased at least one or more systemic medications, while 4.1% did not. The basic characteristics are given in Table 1. Women purchased significantly more medications than men and were also significantly more likely to have a hospital diagnosis before 1985. Significantly more men than women had periodontitis at baseline, but no significant difference in tobacco use between the genders was recorded.

**Table 1:** Basic characteristics of the participants.

Characteristics	Total	Women	Men	*p* value
Participants	1495 (100%)	765 (51.2%)	730 (48.8%)	
Medication purchases above the median	735 (100%)	421 (57.3%)^a^	314 (42.7%)^a^	< 0.001
Dental appointments above the median	755 (100%)	400 (53.6%)^a^	355 (46.4%)^a^	0.157
Tobacco consumption in 1985	518 (100%)	271 (52.3%)^a^	247 (47.7%)^a^	0.519
Hospital diagnosis before 1985	519 (100%)	319 (61.5%)^a^	200 (38.5%)^a^	< 0.001
Periodontitis in 1985	243 (100%)	107 (44.0%)^a^	136 (46.0%)^a^	0.015
Lower socioeconomic status in 1985	292 (100%)	117 (40.1%)^a^	155 (59.9%)^a^	0.105

^a^ Number of participants and percentages within the given row.

Table 2 gives details of the dental appointments, treatments, and medication purchases registered. Between the years 2008 and 2017, 92.2% of our participants attended at least one dental appointment. The most common reasons for dental appointments and respective treatments were dental examinations and placing fillings. As to the purchase of medications, 95.9% did purchase at least one medication. The median of all medication purchases during the time of observation was 85, equivalent to 6.5 medication purchases a year. In the 12 years of the pharmacy register follow‐up from 2005 to 2017, 90.4% of our patients had purchased different medications from more than one ATC subgroup. The most purchased ATC main groups and subgroups of medications are also given in Table 2. The median for all the other subgroups not reported here was 0 (see Attachment S1).

**Table 2:** Means and medians for the most purchased ATC main groups and subgroups of medications during the years 2005–2017, as well as dental appointments and treatments during the years 2008–2017.

	*N*	Mean (SD)	Median
Dental appointments	1378	36.0 (24.9)	33
Dental exam	1373	12.3 (8.1)	12
Fillings	1263	6.4 (6.8)	5
Periodontal treatment	1123	5.5 (5.9)	4
X‐ray	1068	2.6 (2.9)	2
Extractions	620	0.9 (1.8)	0
Root canal treatment	469	0.8 (1.5)	0
Saliva test	9	0.01 (0.1)	0
All types of medications	1434	146.1 (202.2)	85
Most purchased main ATC groups of medications			
Anti‐infectives for systemic use	1287	8.4 (22.4)	4
Nervous system	1111	36.9 (126.4)	4
Respiratory system	1083	12.6 (32.6)	3
Musculoskeletal system	1080	7.2 (17.8)	2
Alimentary tract and metabolism	1024	20.9 (50.5)	3
Cardiovascular system	937	34.2 (52.9)	5
Most purchased ATC subgroups of medications			
Antibacterials for systemic use	1272	5.6 (8.1)	3
Anti‐inflammatory and antirheumatic products	1029	4.8 (9.1)	2
Analgesics	937	8.9 (27.8)	1
Ophthalmological	642	4.7 (17.0)	0
Corticosteroids, dermatological preparations	621	2.1 (5.8)	0
Psycholeptics	614	12.6 (56.3)	0
Drugs for constipation	602	2.2 (7.1)	0
Nasal preparations	601	2.3 (6.8)	0
Cough and cold preparations	598	1.6 (5.5)	0
Drugs for acid‐related disorders	578	4.6 (14.4)	0

Table 3 gives the number of medication purchases concerning the number of dental appointments. The results show that patients who had a high number of purchases of systemic medications had significantly more dental appointments than those with a low number of purchases. This was observed for all types of dental treatments. Only nine saliva tests were documented. These were made entirely on patients who had a high number of purchases of medications (*p* = 0.002).

**Table 3:** Chi‐square tests for differences between high and low purchasers regarding different variables.

Variable (higher dental appointments)	High medication purchasers (%)	Low medication purchasers (%)	χ^2^	*p* value
All dental appointments	56.3	43.7	31.00	< 0.001
Dental examinations	54.1	45.9	14.86	< 0.001
Radiographs	57.1	42.9	24.20	< 0.001
Periodontal treatments	51.9	48.1	4.01	0.045
Fillings	53.9	46.1	13.69	< 0.001
Extractions	55.0	45.0	14.44	< 0.001
Root canal treatment	56.9	43.1	16.49	< 0.001

In the table are Chi‐square test of the distribution of data on four cells but only two cells are shown, for a complete distribution contact the first author. The degree of freedom of all variables are 1.

Table 4 gives a comprehensive overview of high and low purchasers of the systemic medications recorded and the number of dental appointments. When assessing the difference between high and low purchasers of medication regarding the number of dental appointments, a significant difference was noted in 12 out of 13 of the analyzed main ATC groups, and 32 out of 96 medication ATC subgroups, respectively. However, a similar trend was seen in all medication types. Not only known xerogenic medications were observed here but also drugs for the treatment of systemic inflammatory diseases and even purchasing flu medications were found in the analyses.

**Table 4:** *t*‐test for differences between high and low number of dental appointments regarding medication purchases related to systemic inflammation.

Variable (higher number of purchases)	High number of dental appointments (%)	Low number of dental appointments (%)	χ^2^	*p* value
All types of medications above median	57.8	42.2	31.00	< 0.001
Alimentary tract and metabolism	56.2	43.8	17.19	< 0.001
Antidiarrheals, intestinal, anti‐inflammatory/anti‐infective agents	60.4	39.6	8.19	0.004
Drugs used in diabetes	55.2	44.8	1.31	0.252
Cardiovascular system	55.2	44.8	12.935	< 0.001
Cardiac therapy	51.5	48.5	0.09	0.762
Antihypertensives	46.4	53.6	0.19	0.663
Diuretics	53.6	46.4	1.49	0.222
Vasoprotectives	61.5	38.5	9.76	0.002
Beta blocking agents	55.8	44.2	7.03	0.008
Calcium channel blockers	54.5	45.5	2.98	0.084
Agents acting on the renin‐angiotensin system	55.4	44.6	8.76	0.003
Lipid modifying agents	56.3	43.8	9.35	0.002
Dermatologicals	60.4	39.6	53.21	< 0.001
Antipsoriatics	56.9	43.1	0.86	0.355
Corticosteroids, dermatological preparations	58.9	41.1	30.24	< 0.001
Systemic hormonal preparations, excl. sex hormones and insulins	57.9	42.1	17.04	< 0.001
Corticosteroids for systemic use	56.9	43.1	9.15	0.002
Thyroid therapy	60.8	39.2	7.99	0.005
Anti‐infectives for systemic use	60.0	40.0	40.94	< 0.001
Antibacterials for systemic use	59.8	40.2	44.20	< 0.001
Antineoplastic and immunomodulating agents	56.0	44.0	1.90	0.168
Antineoplastic agents	71.9	28.1	5.98	0.015
Musculo‐skeletal system	54.8	45.2	9.68	0.002
Anti‐inflammatory and antirheumatic products	55.7	44.3	11.02	< 0.001
Nervous system	54.3	45.7	8.06	0.005
Anesthetics	67.2	32.8	7.45	0.006
Analgesics	54.8	45.2	11.15	< 0.001
Antiepileptics	58.2	41.8	2.80	0.094
Anti‐parkinson drugs	58.5	41.5	1.089	0.297
Psycholeptics	53.6	46.4	3.96	0.047
Psychoanaleptics	54.4	45.6	2.93	0.087
Other nervous system drugs	55.4	44.6	0.95	0.329
Respiratory system	57.5	42.5	24.05	< 0.001
Drugs for obstructive airway diseases	55.2	44.8	4.420	0.036
Antihistamines for systemic use	54.8	45.2	5.03	0.025

The table shows the chi‐square test of the distribution of data on four cells, but only two cells are shown. For a complete distribution, contact the first author. The degree of freedom of all variables is 1.

In addition to the ATC main groups and their subgroups given in Table 4, significant differences were found regarding the higher number of medication purchases versus the number of dental appointments in other subgroups of medications. These ATC groups included the main group of genito‐urinary system and sex hormones (*n* = 375, *p* = 0.003), the subgroup of sex hormones and modulators of the genital system (*n* = 264, *p* = 0.010), medications for blood and blood‐forming organs (*n* = 388, *p* < 0.001), and drugs for anemia (*n* = 176, *p* < 0.001). Further, significant differences were also observed in the purchase of drugs for sensory organs (*n* = 343, *p* < 0.001), and ophthalmological and otological preparations (*n* = 214, *p* < 0.001). Antiparasitic products, insecticides, and repellents (*n *= 336, *p* < 0.001), and antiprotozoals (*n* = 214, *p* < 0.001), also showed significant differences with a higher number of purchases in the group with a higher number of dental appointments. For flu medications, a significant difference could be seen for nasal preparations (*n *= 340, *p* < 0.001) and cough and cold preparations (*n* = 457, *p* < 0.001). Among the gastrointestinal medications, antacids (*n *= 314, *p* = 0.019), mineral supplements (*n* = 154, *p* = 0.003), and stomatological preparations (*n* = 157, *p* = < 0.001), respectively, were associated with more dental appointments and treatments.

Table 5 reports the logistic regression analysis that was performed to examine the relationship between the frequency of the number of medications purchased and the number of dental appointments. The results showed that purchasing more medications were significantly associated with more dental appointments, even after adjusting for covariables.

**Table 5:** Logistic regression results for associations between medication purchases and dental appointments.

Medication purchase versus dental appointment	Significance	OR (95%)	Cl lower	Cl higher
Dental appointments above the median	< 0.001	1.759	1.425	2.171
Man (sex)	< 00.001	0.672	0.544	0.829
Tobacco consumption in 1985	0.023	1.291	1.036	1.610
Diagnosis before 1985	< 00.001	1.658	1.328	2.070
Low socioeconomic status in 1985	0.017	0.830	0.637	1.083
Dental examinations above the median	< 00.001	1.468	1.191	1.809
Man (sex)	< 00.001	0.674	0.547	0.831
Tobacco consumption in 1985	0.007	1.352	1.087	1.683
Diagnosis before 1985	< 00.001	1.635	1.312	2.039
Low socioeconomic status in 1985	0.0143	0.820	0.630	1.069
Dental radiographs above the median	< 00.001	1.588	1.282	1.966
Man (sex)	< 00.001	0.671	0.544	0.827
Tobacco consumption in 1985	0.010	1.334	1.071	1.661
Diagnosis before 1985	< 00.001	1.564	1.255	1.951
Low socioeconomic status in 1985	0.111	0.807	0.620	1.050
Anti‐infective treatments above the median	0.043	1.243	1.007	1.533
Man (sex)	< 00.001	0.668	0.543	0.824
Tobacco consumption in 1985	0.007	1.352	1.087	1.682
Diagnosis before 1985	< 00.001	1.644	1.318	2.052
Low socioeconomic status in 1985	0.104	0.804	0.612	1.046
Placing fillings above median	< 00.001	1.427	1.160	1.755
Man (sex)	< 00.001	0.671	0.545	0.827
Tobacco consumption in 1985	0.005	1.364	1.096	1.696
Diagnosis before 1985	< 00.001	1.600	1.284	1.994
Low socioeconomic status in 1985	0.085	0.793	0.610	1.032
Extractions above median	< 00.001	1.442	1.167	1.782
Man (sex)	< 00.001	0.655	0.532	0.808
Tobacco consumption in 1985	0.016	1.310	1.052	1.633
Diagnosis before 1985	< 00.001	1.589	1.275	1.981
Low socioeconomic status in 1985	0.067	0.797	0.613	1.038
Root canal treatments above the median	< 00.001	1.500	1.199	1.877
Man (sex)	< 00.001	0.667	0.541	.823
Tobacco consumption in 1985	0.005	1.364	1.096	1.697
Diagnosis before 1985	< 00.001	1.571	1.260	1.958
Low socioeconomic status in 1985	0.110	0.807	0.620	1.050

Significance was set at *p*‐value < 0 0.05.

Table 6 gives the results of the logistic regression analyses on the purchase of medications used for inflammatory diseases versus dental appointments. The associations were significant even when considering all the covariates. Hence, a significant positive association was found between the number of medication purchases and the number of dental appointments. A more frequent number of dental appointments was found in 12 out of 13 of the ATC groups of medications as well as in 32 ATC subgroups of medications, respectively.

**Table 6,:** Logistic regressions for associations between the number of dental appointments and purchases of medications used for treating systemic inflammatory diseases.

Dental visits versus medication main ATC groups and subgroups	Significance	OR (95%)	Cl lower	Cl higher
Alimentary tract and metabolism	< 00.001	1.759	1.425	2.170
Drugs used in diabetes	< 00.001	1.566	1.268	1.933
Antidiarrheals, intestinal, anti‐inflammatory/anti‐infective agents	0.132	1.322	0.919	1.902
Cardiovascular system	0.006	1.577	1.141	1.773
Vasoprotectives	< 00.001	1.451	1.180	1.785
Beta blocking agents	0.001	1.697	1.227	2.346
Agents acting on the renin‐angiotensin system	0.012	1.339	1.065	1.683
Lipid modifying agents	0.004	1.366	1.103	1.690
Dermatological	.003	1.392	1.115	1.739
Antibiotics and chemotherapeutics for dermatological use	< 00.001	2.194	1.777	2.709
Corticosteroids, dermatological preparations	< 00.001	2.013	1.467	2.761
Systemic hormonal preparations, excluding sex hormones and insulins	< 00.001	1.744	1.411	2.155
Corticosteroids for systemic use	< 00.001	1.581	1.265	1.975
Anti‐infectives for systemic use	0.005	1.642	1.166	2.313
Antibacterials for systemic use	< 00.001	1.986	1.602	2.462
Antineoplastic and immunomodulating agents	< 00.001	3.160	2.292	4.357
Antineoplastic agents	0.202	1.260	.884	1.795
Musculoskeletal system	0.021	2.512	1.149	5.494
Anti‐inflammatory and antirheumatic products	0.002	1.403	1.138	1.729
Nervous system	< 00.001	1.871	1.490	2.351
Anesthetics	0.009	1.323	1.072	1.633
Analgesics	0.022	1.882	1.096	3.230
Respiratory system	< 00.001	1.579	1.272	1.959
Drugs for obstructive airway diseases	< 00.001	1.685	1.362	2.086
Antihistamines for systemic use	0.048	1.276	1.003	1.624

Covariates included in the analysis were the ATC main and subgroups for medications, sex, tobacco consumption in 1985, a periodontal diagnosis in 1985, hospital diagnosis before 1985 and low socioeconomic status in 1985. Significance was set at *p*‐value < 0 0.05.

## 4 Discussion

The main hypothesis of the present study was that participants purchasing a high number of medications have more dental appointments and treatments when compared with those purchasing fewer medications. Our findings confirmed the hypothesis. Medication purchases above the median posed a higher statistical risk for more dental appointments and, subsequently, presumably indicated poorer oral health. A significant association was indeed found between all the ATC main groups of medications and dental appointments, except for antineoplastic and immunomodulating agents. A significant association was also found for over a third of the 2nd‐level ATC subgroups of medications. These findings are in line with earlier research on polypharmacy and comorbidities that have been associated with low salivary flow and subsequent dry mouth in the elderly (Smidt et al. 2010).

Purchasing more vasoprotective drugs, beta‐blocking agents, medicines acting on the renin‐angiotensin system, and lipid‐modifying agents, was associated with a significantly higher number of dental appointments and treatments. Indeed, many medications have been linked to hyposalivation, and, in particular, cardiovascular drugs are known to be xerogenic, thus affecting oral health (Schenkein et al. 2020). Whether the observed polypharmacy and a higher medication purchase of almost all medication groups indicate a higher risk for worse oral health nevertheless calls for more studies. Heterogeneity among studies on dry mouth and the role of polypharmacy in it still poses a challenge for evidence (Wiriyakijja et al. 2023).

The long‐term follow‐up of our patients offered an insight into how long‐term oral health, systemic health, medication use, and socioeconomic status affect a patient. The registers at hand were extensive. The pharmacological register used here provided a unique possibility for analyzing the relationships between medication purchases and the number of dental appointments, which is a strength in our study. However, the longevity of the study also poses challenges, since medication purchases and everyday habits may differ from year to year. These data are missing. Tracking changes in medications is problematic, as the initial treatment does not necessarily suit the patient thus requiring a change both in the medication type and dosage. This may even lead to changes in the ATC medication groups, something that could not be tracked in our study. Furthermore, in the registers at hand, one could not follow smoking, snus, or nicotine product use throughout the decades, which is a weakness. Oral health habits and alcohol use were not included in the registers, which also was unfortunate.

Periodontitis has been associated with systemic chronic inflammatory comorbidities (Hajishengallis and Chavakis 2021). Notable conditions are cardiovascular diseases, diabetes, inflammatory bowel diseases, gastrointestinal and colorectal cancer, neurodegenerative diseases, rheumatic diseases, adverse pregnancy outcomes, as well as respiratory tract infections (Herrera et al. 2024; Bui et al. 2019). In our study, a significant association was observed between the number of dental appointments and the number of purchases of the drugs used for systemic inflammatory disease. Yet, when assessing for confounders, periodontitis at baseline was not significantly associated with the purchase of medicines later in life. On the other hand, hospital diagnosis before baseline in 1985 was a significant “protective factor” for most ATC groups and subgroups of medications. Associations of systemic corticosteroids, anti‐inflammatory medications, and anti‐infectives for systemic and dermatological use with more dental appointments were also found. Antibiotics contain a broad range of drugs such as tetracyclines, beta‐lactam antibacterials, sulfonamides, trimethoprim, and macrolides. Penicillin, metronidazole, cefalexin, and clindamycin, are antibiotics also used in dentistry since oral pathogens have been linked to a higher risk of infections (Han and Wang 2013). Anti‐virals, such as acyclovir, are used for treating viral infections such as herpes lesions, which also can lead to more dental appointments. Earlier research suggests a connection between periodontitis, viruses, and comorbidities (Teles et al. 2022). In our study, even cold and flu medication purchases were associated with more dental appointments. These medications can also cause dry mouth. On the other hand, pericoronitis, for example, has earlier been linked to more flu episodes among men doing their military service in Finland (Meurman et al. 1995), so there seems to be a bidirectional relationship even here.

Antineoplastic agents are a very diverse pharmacological group. A significant association between this ATC main group and the number of dental appointments could not be established. However, when analyzing the largest ATC subgroup of neoplastic agents, a significant association was found. Neurological and psychiatric diseases such as Alzheimer's, Parkinson's, and depression, have also been associated with periodontitis (Liccardo et al. 2019; Chen et al. 2017). In the present study, more dental appointments were significantly associated with purchasing more than the median of the nervous system drugs in general. However, a significant association between purchasing antiepileptics, antiparkinson drugs, or psycholeptics and the number of dental appointments could not be found. An association between analgesics and anesthetics could, however, be found. As these drugs are routinely used also in dentistry, the result was not unexpected.

Drugs for obstructive airway diseases are known to be associated with periodontitis, but asthma medications are also associated with a higher risk of oral candidiasis and caries (Moreira et al. 2023; Thomas et al. 2010). In addition, purchasing ophthalmological drugs was associated with more dental appointments. This might explain that both the daily intake of many drugs and systemic diseases themselves link to both oral and ocular dryness (Smidt et al. 2011). In addition, another possible link can be speculated because impaired vision may affect oral healthcare and consequently reflects in the higher need for dental treatments (Blanco López et al. 2023).

Furthermore, diabetes is associated with periodontitis and the risk of caries (Larvin et al. 2023). However, in our study, the lack of a significant association between the number of dental appointments and purchases of diabetes medications was surprising. Socioeconomic status, in turn, has been linked to disease development, medication purchases, worse oral health, and poorer oral health‐related quality of life (Knorst et al. 2021; Iqbal et al. 2023). In our study, low socioeconomic status at baseline negatively correlated with the number of medication purchases and dental appointments above the median. This might be due to the high costs of medications and appointments among the disadvantaged and participants with lesser means.

Some possible causal mechanisms for the observed associations still need to be discussed. These include poor oral hygiene, trauma, tobacco use and diet. In the adjusted odds ratios, which included the covariates like tobacco use and periodontal status at baseline, further mechanisms for example, dietary habits had not been recorded. The eventual causal pathways between medication purchases, diet, and oral hygiene thus require further investigation.

To conclude, in the group of adult Swedes who were studied, the present results confirmed our hypothesis by showing that a higher number of purchased medications was associated with the number of dental appointments and treatments. Since most of the medication ATC main groups and subgroups, and especially the drugs used for systemic inflammatory diseases, were associated with more dental appointments and treatments, the result indicates that not only anticholinergic drugs but simply polypharmacy and higher medication use in general potentially affects oral health negatively. This leads to an increased need for dental appointments. This is in line with the paradigm that oral health is connected to systemic health.

## Author Contributions

Birgitta Söder and Jukka Meurman contributed to the conception, design, data acquisition, and interpretation, and critically revised the manuscript. Håkan Källmén and Esa Korpi contributed to data analysis and interpretation and critically revised the manuscript. Freja Frankenhaeuser conducted the main part of the analyses, contributed to the design and data interpretation and drafted the manuscript. All authors gave final approval and agreed to be accountable for all aspects of the work.

## Ethics Statement

The Ethics Committee of the Karolinska University Hospital at Huddinge had approved the study (Dnr 2007/1669–31; 2012/590–32; 2017/2204–32).

## Consent

This is a register study. Written informed consent was not required.

## Conflicts of Interest

The authors declare no conflicts of interest.

## Supporting information

Attachment 1. Complete list of means and medians for all ATC main groups and subgroups of medication purchases.

## Data Availability

The data that support the findings of this study are available on request from the corresponding author. The data are not publicly available due to privacy or ethical restrictions.
