# Supplementary material for: Medication Purchases Are Associated With the Number of Dental Treatments
Source: Clin Exp Dent Res. 2025 May 23;11(3):e70121. doi: 10.1002/cre2.70121 (PMC12102072; doi:10.1002/cre2.70121)
Supplement: Supplementary file 1 — Attachment 1. Complete list of means and medians for all ATC main groups and subgroups of medication purchases. [file CRE2-11-e70121-s001.docx]

Attachment 1. Complete list of means and medians for all ATC main groups and subgroups of medication purchases

| ATC main and subgroups | | N | Mean (SD) | Median |
| --- | --- | --- | --- | --- |
| All types of medications (ATC) | | 1434 | 146.1 (202.2) | 85 |
| Alimentary tract and metabolism A | | 1024 | 20.9 (50.5) | 3 |
|  | Stomatological preparations A01 | 242 | 0.8 (3.7) | 0 |
|  | Drugs for acid related disorders A02 | 578 | 4.6 (14.4) | 0 |
|  | Drugs for functional gastrointestinal disorders A03 | 171 | 0.6 (5.2) | 0 |
|  | Antiemetics and antinauseants A04 | 48 | 0.1 (0.7) | 0 |
|  | Biler and liver therapy A05 | 7 | 0.1 (1.7) | 0 |
|  | Drugs for constipation A06 | 602 | 2.2 (7.1) | 0 |
|  | Antidiarrheals, intestinal, antiinflammatory/antiinfective agents A07 | 182 | 1.0 (6.4) | 0 |
|  | Antiobesity preparations A08 | 50 | 0.3 (2.8) | 0 |
|  | Digestives A09 | 24 | 0.2 (3.2) | 0 |
|  | Drugs used in diabetes A10 | 134 | 3.4 (15.4) | 0 |
|  | Vitamins A11 | 183 | 1.6 (11.6) | 0 |
|  | Mineral supplements A12 | 261 | 2.1 (6.9) | 0 |
|  | Anabolic agents for systemic use A14 | 1 | 0.0 (0.1) | 0 |
|  | Other alimentary tract and metabolism products A16 | 2 | 0.0 (0.1) | 0 |
| Blood and blood forming organ B | | 700 | 10.2 (30.0) | 0 |
|  | Antithrombotic agents B01 | 538 | 6.2 (16.8) | 0 |
|  | Antihemorrhagics B02 | 26 | 0.0 (0.5) | 0 |
|  | Antianemic preparation B03 | 291 | 3.0 (13.0) | 0 |
|  | Blood substitutes and perfusion solutions B05 | 5 | 0.1 (4.0) | 0 |
| Cardiovascular system C | | 937 | 34.2 (52.9) | 5 |
|  | Cardiac therapy C01 | 200 | 1.4 (10.3) | 0 |
|  | Antihypertensives C02 | 28 | 0.3 (5.0) | 0 |
|  | Diuretics C03 | 302 | 3.6 (14.4) | 0 |
|  | Vasoprotectives C05 | 179 | 0.3 (1.7) | 0 |
|  | Beta blocking agents C07 | 437 | 7.1 (17.4) | 0 |
|  | Calcium channel blockers C08 | 356 | 4.5 (14.4) | 0 |
|  | Agents acting on the renin-angiotensin system C09 | 569 | 9.8 (18.7) | 0 |
|  | Lipid modifying agents C10 | 480 | 7.3 (18.0) | 0 |
| Dermatologicals D | | 895 | 7.0 (18.6) | 1 |
|  | Antifungals for dermatological use D01 | 459 | 1.0 (2.8) | 0 |
|  | Emollients and protectives D02 | 378 | 2.2 (8.5) | 0 |
|  | Antipruritics A04 | 22 | 0.0 (0.4) | 0 |
|  | Antipsoriatics D05 | 51 | 0.2 (2.3) | 0 |
|  | Antibiotics and chemotheraputics for dermatological use D06 | 198 | 0.3 (1.9) | 0 |
|  | Corticosteroids, dermatological preparations D07 | 621 | 2.1 (5.8) | 0 |
|  | Antispetics and disinfectants D08 | 22 | 0.0 (0.2) | 0 |
|  | Medicated dressings D09 | 6 | 0.0 (0.3) | 0 |
|  | Anti-acne preparations D10 | 82 | 0.3 (2.1) | 0 |
|  | Other dermatological preparations D11 | 64 | 0.1 (0.9) | 0 |
| Genito urinary system and sex hormones G | | 775 | 11.7 (22.3) | 1 |
|  | Gynecological antiinfectives and antiseptics G01 | 86 | 0.1 (0.8) | 0 |
|  | Other gynecologicals G02 | 33 | 0.1 (1.4) | 0 |
|  | Sex hormones and modulators of the genital system G03 | 477 | 6.4 (14.2) | 0 |
|  | Urologicals G04 | 350 | 3.1 (10.9) | 0 |
| Systemic hormonal preparations, excl. sex hormones and insulins H | | 516 | 5.8 (15.7) | 0 |
|  | Pituitary and hypothalmic hormones and analogues H01 | 14 | 0.1 (2.5) | 0 |
|  | Coticosteroids for systemic use H02 | 404 | 1.5 (6.8) | 0 |
|  | Thyroid therapy H03 | 166 | 3.8 (12.7) | 0 |
|  | Pancreatic hormones H04 | 1 | 0.0 (0.1) | 0 |
|  | Calcium homeostatis H05 | 1 | 0.0 (0.2) | 0 |
| Antiinfectives for systemic use J | | 1287 | 8.4 (22.4) | 4 |
|  | Antibacterials for systemic use J01 | 1272 | 5.6 (8.1) | 3 |
|  | Antimyotics for systemic use J02 | 129 | 0.2 (1.4) | 0 |
|  | Antimycobacterials J04 | 5 | 0.0 (0.3) | 0 |
|  | Antivirals for systemic use J05 | 170 | 1.1 (9.2) | 0 |
|  | Immune sera and immunoglobulins J06 | 4 | 0.0 (0.8) | 0 |
|  | Vaccines J07 | 125 | 0.1 (0.7) | 0 |
| Antineoplastic and immunomodulating agents L | | 141 | 3.2 (21.8) | 0 |
|  | Antineoplastic agents L01 | 32 | 0.1 (1.7) | 0 |
|  | Endocrine therapy L02 | 54 | 0.5 (2.8) | 0 |
|  | Immunostimulants L03 | 20 | 0.1 (1.7) | 0 |
|  | Immunosupressants L04 | 49 | 1.0 (8.3) | 0 |
| Musculo-skeletal system M | | 1080 | 7.2 (17.8) | 2 |
|  | Antinflammatory and antirheumatic products M01 | 1029 | 4.8 (9.1) | 2 |
|  | Topical products for joint and muscular pain M02 | 159 | 0.2 (1.1) | 0 |
|  | Muscle relaxants M03 | 163 | 0.7 (8.7) | 0 |
|  | Antigout preparations M04 | 56 | 0.4 (3.1) | 0 |
|  | Drugs for treatment of bone disease M05 | 73 | 0.5 (3.5) | 0 |
|  | Other drugs for disorders of the musculo-skeletal system M09 | 9 | 0.0 (0.2) | 0 |
| Nervous system N | | 1111 | 36.9 (126.4) | 4 |
|  | Anesthetics N01 | 64 | 0.1 (0.6) | 0 |
|  | Analgesics N02 | 937 | 8.9 (27.8) | 1 |
|  | Antiepileptics N03 | 110 | 1.7 (12.5) | 0 |
|  | Anti-parkinson drugs N04 | 41 | 0.8 (14.2) | 0 |
|  | Psycholeptics N05 | 614 | 12.6 (56.3) | 0 |
|  | Psychoanaleptics N06 | 362 | 6.3 (22.9) | 0 |
|  | Other nervous system drugs N07 | 92 | 0.6 (12.2) | 0 |
| Antiparasitic products, insecticides and repellents P | | 336 | 0.7 (3.7) | 0 |
|  | Antiprotozoals P01 | 325 | 0.5 (2.3) | 0 |
|  | Anthelmintics P02 | 8 | 0.0 (0.1) | 0 |
|  | Ectoparasiticides P03 | 9 | 0.0 (0.1) | 0 |
| Respiratory system R | | 1083 | 12.6 (32.6) | 3 |
|  | Nasal preparations R01 | 601 | 2.3 (6.8) | 0 |
|  | Thorat preparations R02 | 2 | 0.0 (0.0) | 0 |
|  | Drugs for obstructibe airway diseases R03 | 375 | 4.4 (17.6) | 0 |
|  | Cough and cold preparations R05 | 598 | 1.6 (5.5) | 0 |
|  | Antihistamines for systemic use R06 | 471 | 2.2 (9.3) | 0 |
|  | Other respiratory system products R07 | 1 | 0.0 (0.0) | 0 |
| Sensory organs S | | 793 | 6.4 (22.8) | 1 |
|  | Opthalmologicals S01 | 642 | 4.7 (17.0) | 0 |
|  | Otologicals S02 | 99 | 0.2 (0.9) | 0 |
|  | Ophtalmological and otological preparations S03 | 356 | 0.6 (1.8) | 0 |
